# Effects of physical activity on the severity of illness and mortality in COVID-19 patients: A systematic review and meta-analysis

**DOI:** 10.3389/fphys.2022.1030568

**Published:** 2022-11-08

**Authors:** Nuttawut Sittichai, Nichapa Parasin, Surasak Saokaew, Sukrit Kanchanasurakit, Nuttawan Kayod, Ketnapa Praikaew, Pochamana Phisalprapa, Mujalin Prasannarong

**Affiliations:** ^1^ Program in Physical Education, Faculty of Education, Phuket Rajabhat University, Phuket, Thailand; ^2^ Division of Physical Therapy, School of Allied Health Science, University of Phayao, Phayao, Thailand; ^3^ Unit of Excellence on Clinical Outcomes Research and IntegratioN (UNICORN), School of Pharmaceutical Sciences, University of Phayao, Phayao, Thailand; ^4^ Center of Health Outcomes Research and Therapeutic Safety (Cohorts), School of Pharmaceutical Sciences, University of Phayao, Phayao, Thailand; ^5^ Division of Social and Administrative Pharmacy, Department of Pharmaceutical Care, School of Pharmaceutical Sciences, University of Phayao, Phayao, Thailand; ^6^ Division of Clinical Pharmacy, Department of Pharmaceutical Care, School of Pharmaceutical Sciences, University of Phayao, Phayao, Thailand; ^7^ Department of Physical Therapy, Faculty of Associated Medical Sciences, Chiang Mai University, Chiang Mai, Thailand; ^8^ Division of Ambulatory Medicine, Department of Medicine, Faculty of Medicine Siriraj Hospital, Mahidol University, Bangkok, Thailand

**Keywords:** exercise, physical activity, SARS-CoV-2, coronavirus, severity, mortality

## Abstract

**Purpose:** This systematic review and meta-analysis investigated the association between Physical activity (PA) before Coronavirus Disease 2019 (COVID-19) infection and the severity of illness and mortality in COVID-19 patients.

**Methods:** A comprehensive search was undertaken to identify retrospective and nonrandomized controlled trial studies comparing the severity and mortality of COVID-19 infection among COVID-19 patients who had previously reported their participation in PA with those who had not. The databases searched were PubMed, Cochrane Library, Scopus, Science Direct, EMBASE, OPENGREY.EU, and ClinicalTrials.gov. The risk of bias was assessed using the Newcastle-Ottawa Scale. A random-effects model was used for determining pairwise meta-analyses. The protocol was registered with PROSPERO (CRD42021262548).

**Results:** Eighteen studies met the inclusion criteria (5 cross-sectional, 12 cohort, and 1 case-control studies). All 1 618 680 subjects were adults. PA significantly decreased the risk of death in COVID-19 patients (odds ratio [OR] 0.34; 95% confidence interval [CI], 0.19–0.62; *p* < 0.001) and the risk of severe outcomes (OR 0.60; 95% CI, 0.48–0.76; *p* < 0.001). Subgroup analysis showed that PA for ≥150 min/wk at a moderate intensity or ≥75 min/wk at a vigorous intensity reduced the risks of severity and mortality. Vigorous PA reduced mortality risk, whereas moderate to vigorous PA reduced the risks of severity and mortality.

**Conclusion:** PA before infection might reduce severity and mortality in COVID-19 patients, especially PA ≥ 150 min/wk of moderate activity or ≥75 min/wk of vigorous activity. However, careful interpretations should be considered due to the difference in PA patterns and severity definitions among included studies. This finding implies that engaging in regular PA, even in different patterns, has beneficial effects on the severity and mortality of COVID-19 patients.

## Introduction

Coronavirus disease 2019 (COVID-19) is an infectious respiratory illness caused by severe acute respiratory syndrome coronavirus 2 (SARS-CoV-2) (Lai et al., 2020). The World Health Organization declared COVID-19 a global pandemic on March 11, 2020 (World Health Organization, 2020). As at September 2022, over 600 million confirmed cases and over six million deaths were attributed to the virus worldwide (World Health Organization, 2022a). Transmission of the virus can occur through direct contact with the respiratory droplets of an infected person (generated through coughing and sneezing). Individuals can also be infected by touching surfaces contaminated with the virus and then touching their face (eg, the eyes, nose, or mouth) (Halperin, 2021). Approximately 80% of cases are asymptomatic or have mild symptoms, whereas the remainder can be severe and critical, leading to death (Verity et al., 2020) or persistent long COVID (Fahriani et al., 2021; Fajar et al., 2021). During the virus’s rapid spread in the absence of a COVID-19 vaccine, many countries implemented restrictive policies (eg, stay-at-home orders and the closures of parks, gymnasiums, and recreation centers). These policies were highly influential in containing the number of COVID-19 infections and preventing healthcare systems from being overwhelmed (Kharroubi and Saleh, 2020). However, the policies led to a significant increase in physical inactivity (PiA) (Stockwell et al., 2021), which has been considered a risk factor for developing COVID-19 severity and mortality (Centers for Disease Control and Prevention, 2020).

Physical activity (PA) has been defined as any bodily movement produced by skeletal muscle function resulting in energy expenditure (Caspersen et al., 1985). PA in daily life can be categorized as structured and incidental activities (Strath et al., 2013). Structured activities are bodily movements that usually occur during free time to promote health and fitness, such as weight training, jogging, and swimming (Strath et al., 2013). Incidental activities are bodily movements that occur during daily living, such as walking to school, gardening, and washing a car (Strath et al., 2013). The PA Guidelines for Americans recommend that all adults between 18 and 65 years of age engage in at least 30 min of moderate-intensity aerobic PA a day for 5 days a week or at least 20 min of high-intensity aerobic PA a day for 3 days a week (Piercy et al., 2018). Regular PA is recommended as a supplement to help strengthen the immune system to defend against COVID-19 infection (da Silveira et al., 2020). In addition, PA counteracts some noncommunicable diseases, such as obesity, diabetes mellitus, and arterial hypertension that increase the likelihood of COVID-19 patients experiencing severe outcomes (Chandrasekaran and Ganesan, 2020). However, there is insufficient scientific evidence supporting the recommendations of the PA Guidelines for Americans. A previous systematic review and meta-analysis of Rahmati et al. (2022) reported that PA decreased hospitalization, intensive care unit (ICU) admissions, and mortality rates of patients with COVID-19 in all study types. COVID-19 patients with a history of resistance and endurance exercises experience a lower rate of hospitalization and mortality, respectively. Meta-analysis results from a few studies showed lower mortality in low and moderate-vigorous PA (Rahmati et al., 2022). Only one study (Lee et al., 2021) was included to analyze the effects of PA level on ICU admission. It is suggested that further study should be recommended due to the limited number of studies.

The present study aimed to systematically review all available evidence and pooled odds ratios (ORs) adjusted by confounding factors to determine whether regular PA before COVID-19 infection affects the severity of illness and mortality in COVID-19 patients.

## Methods

### Protocol and registration

This investigation was based on the Preferred Reporting Items for Systematic Reviews and Meta-Analyses (PRISMA) guidelines for healthcare applications (Page et al., 2021). The protocol was prospectively registered with PROSPERO (www.crd.york.ac.uk/PROSPERO; reference CRD42021262548).

### Information sources and search strategy

The PubMed, Cochrane Library, Scopus, Science Direct, and EMBASE databases were systematically searched through 15 June 2022. The search keywords were “COVID*,” “exercise,” “physical activity,” “mortality,” and “severity” (Supplementary Table S1). In addition, grey literature was searched in OPENGREY.EU and ClinicalTrials.gov through 18 September 2022. No language restrictions were applied. Reference lists were also manually reviewed to find citations for additional pertinent meta-analyses and reviews.

### Eligibility criteria

Each study identified by the search was reviewed (by NS, NK, and KP in the authors’ list) to determine if it had information on the PA or exercise habits of COVID-19 patients before their infection. The PA definition follows WHO (World Health Organization, 2022b), such as regular movement during leisure time, moderate- and vigorous-intensity PA, and active activity (i.e., walking, cycling, wheeling, sports, active recreation, and play). The comparators (physical inactivity; PiA) were activities or habits that were not classified as PA definition of WHO. The primary outcomes of interest were the severity and mortality of COVID-19. Severe COVID-19 follows WHO severity definitions (World Health Organization, 2022c), including severe and critical COVID-19. Articles that reported ORs of severely symptomatic patients admitted to a hospital under treatment (in the general ward and ICU) were included in this study. Non-severe COVID-19 was patients who had an absence of any sign of severe or critical COVID-19. Mortality was confirmed deaths from COVID-19, both inpatient and outpatient, reported in included studies. Moreover, case reports, case series, letters, and studies without interesting outcomes or group comparisons were excluded.

### Study selection

Two investigators (NK and KP in the authors’ list) independently screened the titles and abstracts of the retrieved studies. Full texts were reviewed as necessary. Trials were determined to be eligible for this study based on the inclusion and exclusion criteria mentioned above. A third investigator (MP in the authors’ list) resolved disagreements.

### Data extraction

Two investigators (NS and KP in the authors’ list) independently extracted details from the selected studies and recorded them in a Microsoft Excel spreadsheet. A third investigator (MP in the authors’ list) resolved disagreements. The data extracted were related to the setting, study design, sample size, patient demographics, PA or exercise details, comparators, number of mortalities, and number of patients at each severity level. The authors of the retrieved studies were contacted for missing data by email.

### Risk of bias assessment

Two investigators (NP and MP in the authors’ list) independently evaluated the risk of bias in the included cohort, cross-sectional, and case-control studies using the Newcastle-Ottawa Scale (NOS) (Stang, 2010). A third investigator (SK in the authors’ list) resolved disagreements. The NOS contains three domains: quality of selection, comparability, and outcome. Each study was defined as low, moderate, and high quality when scores were 0–3, 4–6, and 7–10, respectively.

### Quality of evidence

The Grading of Recommendations, Assessment, Development and Evaluation (GRADE) approach was used to rate the quality of evidence of estimates (Guyatt et al., 2008). This study used GRADE to determine the quality of evidence for outcome according to five domains: risk of bias, inconsistency, indirectness, imprecision, and publication bias. The levels of evidence can be categorized into four levels: high, moderate, low, and very low.

### Data synthesis and analysis

Analyses were conducted using Stata Statistical Software, release 14.1 (StataCorp LLC, College Station, TX, United States). A random-effects model was used for determining pairwise meta-analyses. The results are reported as odds ratios (ORs) and 95% confidence intervals (CIs). Heterogeneity in each pairwise comparison was estimated using the I^2^ statistic. Publication bias was assessed using a funnel plot, and Egger’s tests were employed to assess the funnel plot asymmetry. Sensitivity analysis and subgroup analysis by level of PA were performed to evaluate the robustness of the results in determining the severity and mortality of illness in COVID-19 patients.

## Results

### General information

**FIGURE 1:**
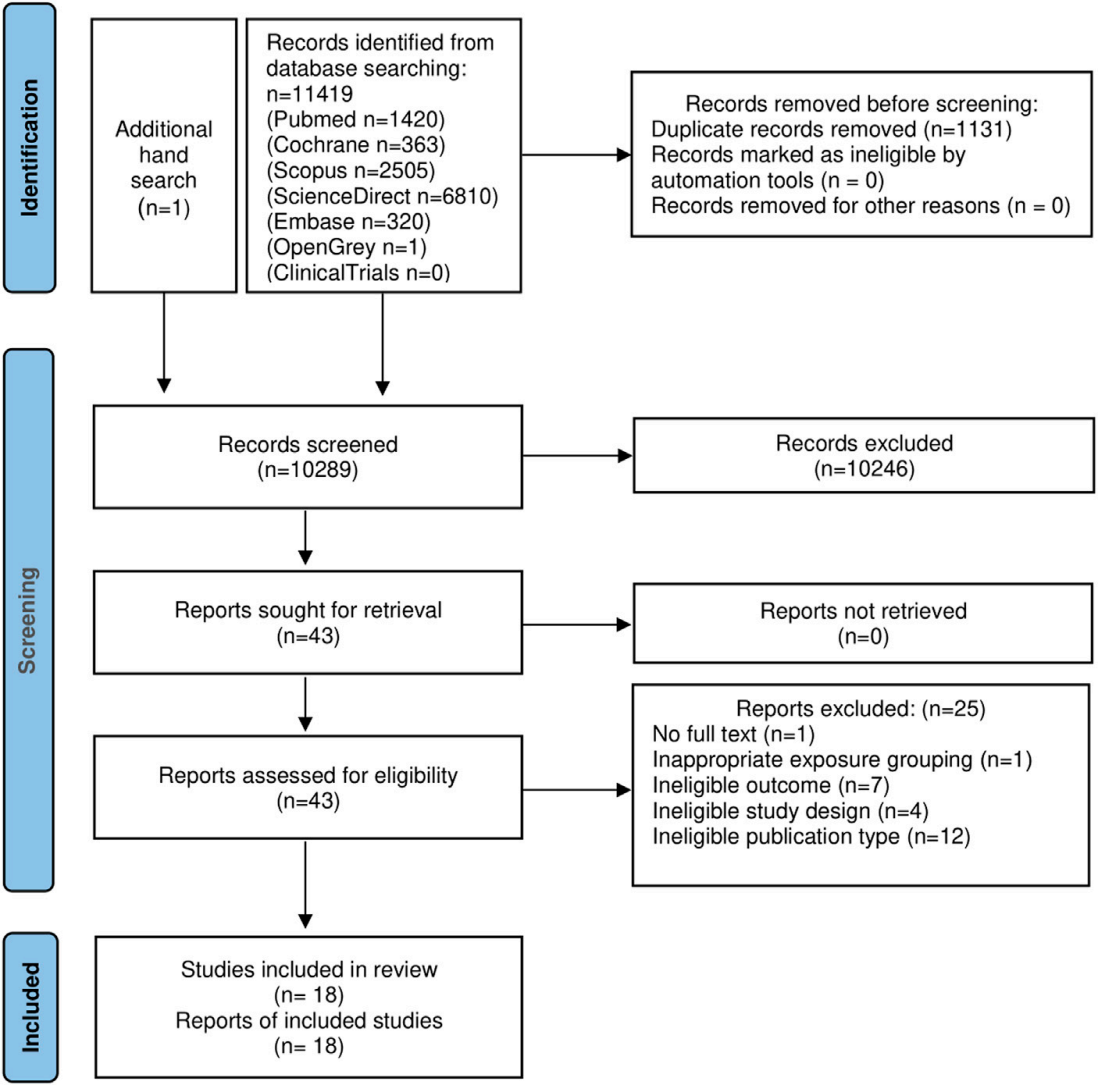
Flow diagram of the literature search in this meta-analysis.

The literature search process is illustrated in Figure 1. The search strategies identified 11 418 articles from five databases and one article from two grey literature databases. Duplicates accounted for 1131 articles and were eliminated. After screening the titles and abstracts, an additional 10 246 articles were excluded for various reasons. Twenty-five articles were excluded from the study after assessment for eligibility (Supplementary Table S2). The meta-analysis was carried out on the remaining 18 eligible original articles.

Table 1 summarizes the core characteristics and outcomes of the selected studies. In all, 1 618 680 subjects were adults. Five studies used cross-sectional designs (Halabchi et al., 2021; Tavakol et al., 2021; Yuan et al., 2021; Tret’yakov et al., 2020; Latorre-Román et al., 2021), 12 had cohort study designs (Sallis et al., 2021; Cho et al., 2021; Lee et al., 2021; Hamrouni et al., 2021; Ahmadi et al., 2021; Hamer et al., 2020; Maltagliati et al., 2021; Malisoux et al., 2022; Baynouna Alketbi et al., 2021; Rowlands et al., 2021; Salgado-Aranda et al., 2021; Pinto et al., 2021), and only one employed a case-control design (Ekblom-Bak et al., 2021). We discovered four studies in the United Kingdom: Hamrouni et al., 2021; Ahmadi et al., 2021; Hamer et al., 2020; and Rowlands et al., 2021. Meanwhile, four studies were conducted in Europe: Latorre-Román et al., 2021; Salgado-Aranda et al., 2021; Ekblom-Bak et al., 2021; and Malisoux et al., 2022. In the meantime, six studies were carried out in Asia (Halabchi et al., 2021; Tavakol et al., 2021; Cho et al., 2021; Lee et al., 2021; Yuan et al., 2021; Baynouna AlKetbi et al., 2021). Besides that, three studies were discovered in the United States (Sallis et al., 2021), Brazil (Pinto et al., 2021), and Russia (Tret’yakov et al., 2020).

**TABLE 1:** Characteristics of the included studies.

Author (year)	Characteristics						
	Setting	Study design	Sample size	Age	Interventions	Comparison	Outcome measure
Halabchi et al. (2021)	Iran	Cross-sectional study	4694	Mean (SD) 42.31 (11.9)	Regular sport (athletes) (Fitness and bodybuilding = 47.8%; Team sport = 16.5%; Combat sports = 19.7%; Individual sports = 16.1%)	Without regular sport (nonathletes)	Death
Sallis et al. (2021)	The United States of America	Cohort study (Retrospective observational study)	48439	Mean (SD) 47.5 (16.97)	Inconsistency active (11–149 min/week) Consistency active (>150 min/week)	Consistency inactive	Death, Severity
Cho et al. (2021)	Korea	Cohort study (Retrospective, nationwide study)	6288	Mean (SD) 50.7 (14.3)	Physical activity moderate to vigorous (using questionnaire comprised three parameters to determine frequency, intensity and metabolic equivalent of task)	Physically inactive	Death
Tavakol et al. (2021)	Iran	Cross-sectional study	206	Mean (SD) 40.9 (11.6)	Moderate to high physical activity level (according by global physical activity questionnaire)	low physical activity level	Severity (Based on the result of symptoms, clinical examinations, and chest radiology) according to clinical classification of COVID-19 released by National Health Commission of China
Tret’yakov et al. (2020)	Russia	Cross-sectional study	298	Median (IQR) 54.5 (44–65	Aerobic exercise during previous 12 months (e.g., running, stationary bike exercise)	Without regular aerobic exercise	Severity
Yuan et al. (2021)	China	Cross-sectional study	164	Mean (SD) 61.8 (13.6)	Based on exercise Vital sign evaluation method (>150 min/week of moderate activity)	Inactivity (<150 min/week of moderate activity or <75 min/week of vigorous activity)	Death, Severity (fever or respiratory infection, plus one of: respiratory rate >30 breaths/min; severe respiratory distress; peripheral capillary oxygen saturation, 93%)
Lee et al. (2021)	South Korea	Cohort study	76395	Not mention	Muscular strengthening >2 times/ week or/and Aerobic physical activity. 150 min/week of moderate intensity or >75 min/week of vigorous intensity activity	Insufficient muscle strengthening activity (<2 times/week) and Insufficient aerobic physical activity (<150 min/week of moderate intensity activity, <75 min/week of vigorous intensity activity and less than an equivalent combination)	Death, Severity
Hamrouni et al. (2021)	The United Kingdom	Cohort study	259397	Median (IQR) Individual who did not covid -19; 68 (61–74) and individual did covid-19; 76 (72–78)	High and moderate physical activity level based on International Physical Activity Questionnaire (IPQA)	Low physical activity level based on IPQA	Death
Ahmadi et al. (2021)	The United Kingdom	Cohort study	468569	Minimum-maximum 40–69	Sufficient physical activity using IPAQ-short form (reported by MET-min/week	Sedentary behavior (low, moderate, high)	Death
Hamer et al. (2020)	The United Kingdom	Cohort study	387109	Mean (SD) 56.2 (8.0)	Sufficient physical activity using IPAQ-short form	Sufficient physical activity	Severity (hospital admission)
Ekblom-bak et al. (2021)	Sweden	Case-control study	279455	Mean (SD) 49.9 (10.7)	Exercise habits (1–2 or >3 times/week)	Exercise habits (never or irregular)	Death, Severity (hospital admission, admission to ICU)
Baynouna Alketbi et al. (2021)	Abu Dhabi (The united Arab Emirates)	a mix retrospective cohort study and case-control study	641	Not mention	Physical activity (times per week)		Death, Severity (ICU admission)
Maltagliati et al. (2021)	European countries	Cohort study	3139	Mean (SD) 69.3 (8.5)	Vigorous physical activity	Low to moderate physical activity	Severity (COVID-19 hospitalization)
Latorre-Roman et al. (2021)	Spain	Cross-sectional study	420	Median (IQR) 33 (20–54)	Moderate physical activity	None physical activity	Severity
Malisoux et al. (2022)	Denmark	Cohort study	452	Median (IQR) 42 (31–51)	Physical active (MET-hour/week)	Sedentary behavior	Severity
Rowlands et al. (2021)	The United Kingdom	Cohort study	82,253	Minimum-maximum 63–68	Moderate to vigorous physical activity (MVPA)		Severity
Salgado-Aranda et al. (2021)	Spain	Cohort study	552	Not mention	Adequate regular exercise (MVPA)	Sedentary or light physical activity	Severity
Pinto et al. (2021)	Brazil	Cohort study	209	Mean (SD) 54.9 (14.5)	Physical activity level consists of 3 sections (work, sport, and leisure-time activity) using the Baecke questionnaire		Severity and death

In terms of focus, 10 studies investigated the impacts of PA on mortality (Ahmadi et al., 2021; Baynouna AlKetbi et al., 2021; Cho et al., 2021; Ekblom-Bak et al., 2021; Halabchi et al., 2021; Hamrouni et al., 2021; Lee et al., 2021; Pinto et al., 2021; Sallis et al., 2021; Yuan et al., 2021), and 14 studies reported the severity of the disease (Tavakol et al., 2021; Yuan et al., 2021; Tret’yakov et al., 2020; Sallis et al., 2021; Lee et al., 2021; Hamer et al., 2020; Maltagliati et al., 2021; Latorre-Román et al., 2021; Malisoux et al., 2022; Ekblom-Bak et al., 2021; Baynouna AlKetbi et al., 2021; Rowlands et al., 2021; Salgado-Aranda et al., 2021; Pinto et al., 2021). Variations in PA or exercise intervention were observed across the 18 studies. Fourteen studies involved PA. In 1 of these 14 studies, one group of participants performed inconsistent activity for between 11 and 149 min/wk, while another group undertook consistent PA for at least 150 min/wk (Sallis et al., 2021). The participants in the 13 other studies engaged in a moderate to high PA level (Hamer et al., 2020; Ahmadi et al., 2021; Baynouna AlKetbi et al., 2021; Cho et al., 2021; Ekblom-Bak et al., 2021; Hamrouni et al., 2021; Latorre-Román et al., 2021; Maltagliati et al., 2021; Pinto et al., 2021; Rowlands et al., 2021; Salgado-Aranda et al., 2021; Tavakol et al., 2021; Malisoux et al., 2022). As for the type of PA, one study reported regular sports, including bodybuilding, team sports, combat sports, and individual sports (Halabchi et al., 2021). However, only three studies showed exercise intervention, including aerobic and muscle strengthening (Yuan et al., 2021; Tret’yakov et al., 2020; Lee et al., 2021).

### Quality assessment

According to the NOS, all studies were rated between 7 and 10 stars. Therefore, all studies were high quality (Table 2). However, the incomplete comparability data in six studies were inadequately explained and not appropriately addressed.

**TABLE 2:** Assessment of quality using the Newcastle-Ottawa Scale.

Study	Selection				Comparability	Outcome			Score
	1	2	3	4	1	1	2	3	
Cross-sectional study									
Halabchi et al. (2021)	*	*	*	**	*	**	*	-	9
Tavakol et al. (2021)	*	*	*	**	**	**	*	-	10
Tret’yakov et al. (2020)	*	*	*	**	*	**	*	-	9
Yuan et al. (2021)	*	*	*	**	**	**	*	-	10
Latorre-Roman et al. (2021)	*		*	**	**	**	*	-	9
Cohort study									
Sallis et al. (2021)	*	*	*	*	**	*	*	*	9
Cho et al. (2021)	*	*	*	*	**	*	*	*	9
Lee et al. (2021)	*	*	*	*	**	*	*	*	9
Hamrouni et al. (2021)	*	*	*	*	**	*	*	*	9
Ahmadi et al. (2021)	*	*	*	*	*	*	*	*	8
Hamer et al. (2020)	*	*	*	*	*	*	*	*	8
Maltagliati et al. (2021)	*	*	*	*	**	*	*	*	9
Salgado-Aranda et al. (2021)	*	*	*	*	*	*	*	*	8
Malisoux et al. (2022)	*	*	*		*	*	*	*	7
Rowlands et al. (2021)	*	*	*	*	**	*	*	*	9
Pinto et al. (2021)	*	*	*	*	*	*	*	*	8
Baynouna Alketbi et al. (2021)	*	*	*	*	**	*	*	*	9
Case control study									
Ekblom-bak et al. (2021)	*	*	*	*	**	*	*	*	9

### Effect of physical activity on severity and mortality

**FIGURE 2:**
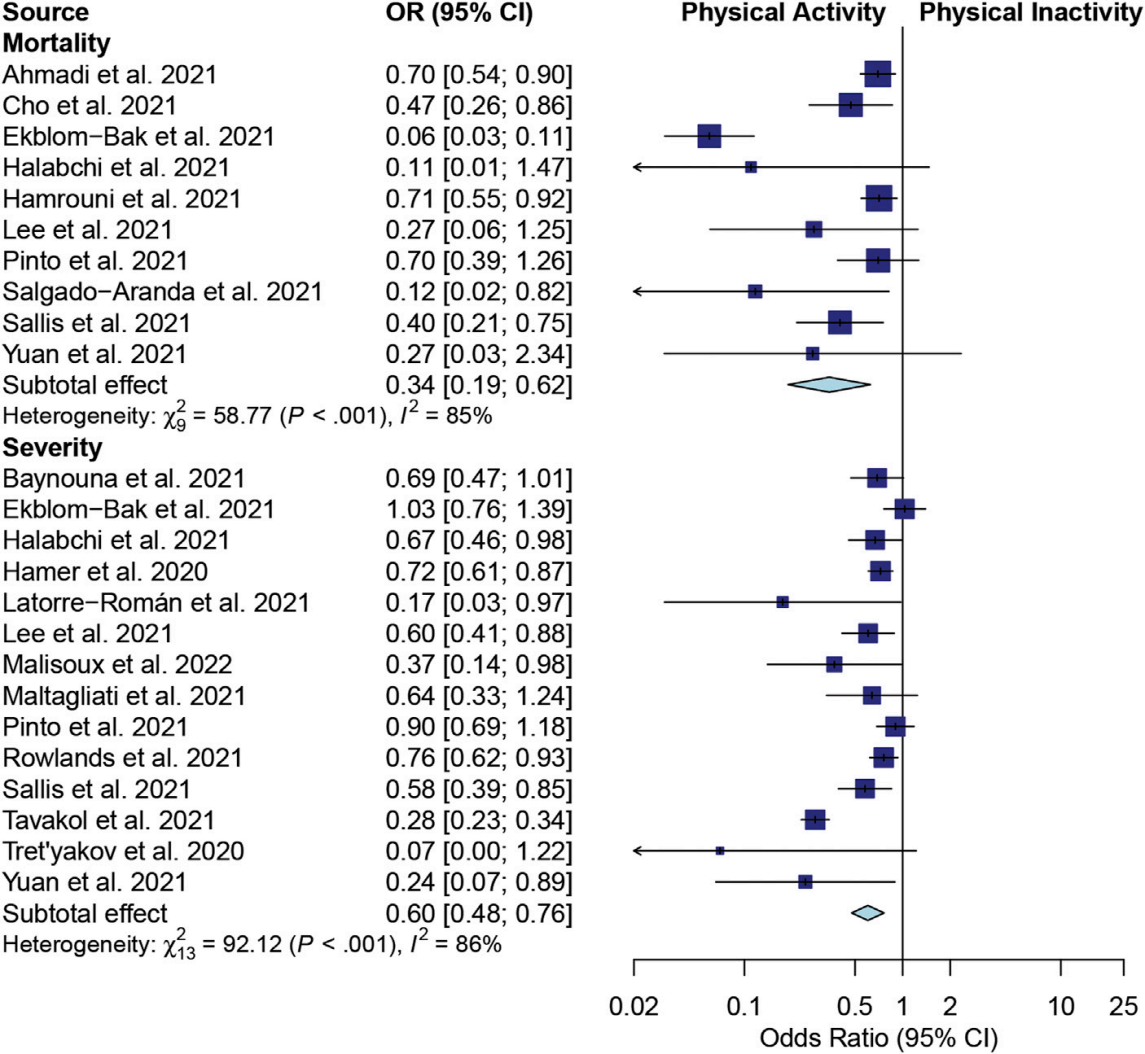
A forest plot of the association between physical activity and mortality and severity in COVID-19 patients.

A pooled OR model was used to evaluate two sets of studies. One set investigated the effects of PA on mortality, and the other set explored PA’s impact on the severity of illness in COVID-19 patients (Figure 2).

Regarding the mortality outcome, 10 studies were meta-analyzed (Ahmadi et al., 2021; Cho et al., 2021; Ekblom-Bak et al., 2021; Halabchi et al., 2021; Hamrouni et al., 2021; Lee et al., 2021; Pinto et al., 2021; Salgado-Aranda et al., 2021; Sallis et al., 2021; Yuan et al., 2021). Significant heterogeneity was found among these articles (I^2^ = 85%; *p* < 0.001). The effects shown as pooled ORs suggested that PA significantly reduced the risk of death in COVID-19 patients compared with PiA (OR 0.34; 95% CI, 0.19–0.62; *p* < 0.001) (Figure 2). A statistical study of the publication bias showed no obvious bias or significant difference in the publications, which was confirmed by a funnel plot (Supplementary Figure S1). The evidence of Egger’s test for bias showed *p* = 0.083 (Supplementary Figure S2).

As for the severity outcome, 14 studies estimated the effects of PA on the severity of disease in patients with COVID-19 (Halabchi et al., 2021; Tavakol et al., 2021; Yuan et al., 2021; Tret’yakov et al., 2020; Sallis et al., 2021; Lee et al., 2021; Hamer et al., 2020; Maltagliati et al., 2021; Latorre-Román et al., 2021; Malisoux et al., 2022; Ekblom-Bak et al., 2021; Baynouna AlKetbi et al., 2021; Rowlands et al., 2021; Pinto et al., 2021). Significant heterogeneity was observed among these articles (I^2^ = 86%; *p* < 0.001). The overall effects of the pooled ORs demonstrated a significantly decreased risk of severe disease in COVID-19 patients with PA compared with PiA (OR 0.60; 95% CI, 0.48–0.76; *p* < 0.001) (Figure 2). As assessed by the funnel plot (Supplementary Figure S1), there was no apparent systematic bias (Egger’s test for bias: *p* = 0.472; Supplementary Figure S2).

### Assessment of quality of evidence

The quality of evidence of the observational studies included in the meta-analysis is determined in Table 3. For each outcome, the observational studies were rated very low on the GRADE scale. Since the included studies were noted to have high heterogeneity, we decided to rate down for the inconsistency. The interventions were delivered in different activities among these studies. Thus, we downgraded the rating of indirectness.

**TABLE 3:** The quality of evidence of the observational studies included for meta-analysis.

Outcome	Quality assessment							Odds ratio (95% CI)	Quality
	Number of studies	Study design	Risk of bias	Inconsistency	Indirectness	Imprecision	Other considerations		
Mortality	10	Observational studies	Not serious^a^	Serious^b^	Serious^c^	Not serious^d^	None^e^	0.34 (0.19–0.62)	⊕ O O O Very low
Severity	14	Observational studies	Not serious^a^	Serious^b^	Serious^c^	Not serious^d^	None^e^	0.60 (0.48–0.76)	⊕ O O O Very low

^a^ Studies were low risk of bias; Newcastle-Ottawa Scale ≥7.

^b^ Inconsistency explained by I^2^ value as more 75%; high heterogeneity; serious.

^c^ The interventions were delivered in different activity among these studies.

^d^ The confidence interval includes benefit from intervention.

^e^ Publication bias is not likely.

### Sensitivity and subgroup analyses

A sensitivity analysis was undertaken to investigate the robustness of the effect estimates. The different models (fixed-effects and random-effects) did not significantly modify the pooled ORs for all outcomes. However, as there were studies on PA at different intensity levels, we performed a subgroup analysis by moderate and high/vigorous intensity. Moreover, some studies identified a sufficient PA level as a PA of ≥150 min/wk of moderate activity or ≥75 min/wk of vigorous activity (Table 4). A subgroup analysis was therefore performed by using sufficient PA. The results showed that PA for ≥150 min/wk at a moderate intensity or ≥75 min/wk at a vigorous intensity reduced the risks of severity and mortality. High or vigorous PA levels reduced the mortality risk, whereas moderate to high/vigorous PA reduced the risks of severity and mortality in COVID-19 patients.

**TABLE 4:** Subgroup analyses of the included studies.

Subgroup analysis by	Number of study	OR (95%CI)	Heterogeneity I^2^ (p-value)	Z (p-value)	List of studies
Mortality					
Moderate to high/vigorous PA	2	0.28 (-0.06–0.63)	44.4% (0.180)	1.84 (0.066)	Cho et al. (2021)
					Salgado-Aranda et al. (2021)
High/vigorous PA	2	0.57 (0.25–0.89)	36.4% (0.210)	2.43 (0.015)	Cho et al. (2021)
					Hamrouni et al. (2021)
Sufficient PA	3	0.56 (0.31–0.81)	37.2% (0.204)	2.50 (0.012)	Sallis et al. (2021)
					Ahmadi et al. (2021)
					Yuan et al. (2021)
Severity					
Moderate to high/vigorous PA	5	0.54 (0.21–0.87)	94.3% (<0.001)	2.10 (0.036)	Latorre-Román et al. (2021)
					Ekblom-Bak et al. (2021)
					Rowlands et al. (2021)
					Tavakol et al. (2021)
					Malisoux et al. (2022)
Sufficient PA	5	0.45 (0.21–0.70)	52.9% (0.075)	2.90 (0.004)	Hamer et al. (2020)
					Tret’yakov et al. (2020)
					Yuan et al. (2021)
					Latorre-Román et al. (2021)
					Sallis et al. (2021)

“Sufficient PA” is defined as the performance of PA for ≥150 min/wk of moderate activity or ≥75 min/wk of vigorous activity. Abbreviations: CI, confidence interval; OR, odds ratio; PA, physical activity.

## Discussion

This systematic review and meta-analysis revealed an association between regular PA and severity and mortality in COVID-19 patients. We included adjusted ORs that considered confounding factors possibly related to the outcomes. The major finding was that regular PA reduced patients’ risks of severity and mortality.

SARS-CoV-2 enters the host cell *via* the angiotensin-converting enzyme (ACE) two receptor. The targeted cells, especially lung parenchyma, have an off-balance between the ACE2/Ang (1–7)/Mas axis and ACE/AngII/AT1R axis that aggravates tissue injury (Yan et al., 2020). Previous studies reported that treatments with ACE inhibitors and angiotensin II receptor blockers were associated with a lower risk of death in COVID-19 patients (Kai and Kai, 2020; Yan et al., 2020; Zhang et al., 2020). Tavakol et al. (2021) reported a reverse correlation between increased PA and COVID-19 disease severity. Our study found a significantly lower risk of mortality with high levels of PA than with PiA. Moderate to high intensity tended to reduce the risk. Cho et al. (2021) reported that dose-dependent exercise reduced infection risk and mortality in COVID-19 patients. They found that vigorous and moderate intensities of exercise training (>1000 metabolic equivalent of task (MET)-min/wk) could reduce the risk of COVID-19 infection and mortality (Cho et al., 2021).

However, excessive exercise has been reported to increase the risk of respiratory infection. Long-term hyperventilation in untrained subjects may involve physical damage to bronchial and alveolar epithelial cells (Nieman, 2000; Murphy et al., 2008). The soluble ACE2 (sACE2) and the transmembrane ACE2 (tACE2) competitively bind with the SARS-CoV-2 virus. Therefore, the probability of a virus entering the targeted cells may be reduced (Yan et al., 2020). However, COVID-19 severity has an opposite response to moderate- and high-intensity exercise. Moderate-intensity exercise increases sACE2, whereas high-intensity exercise increases tACE2 (Hagiu, 2021). Our study found a reduced severity risk in patients who previously had sufficient PA (≥150 min/wk of moderate activity or ≥75 min/wk of vigorous activity) or moderate to high-intensity PA.

Regular exercise can improve the systemic immune response and reduce infection. The ratio of natural killer (NK) cells is modified, and changes in T helper cell expression and function due to exercise may improve the body’s resistance to respiratory infections (Timmons and Cieslak, 2008; Nieman and Wentz, 2019). Moderate exercise increases NK-cell activity (Nieman et al., 1990; Nieman, 2000). In addition, T helper (Th) cells (Th1 and Th2) and T-cell function in the upper respiratory tract mucosa are improved (Timmons and Cieslak, 2008; Nieman and Wentz, 2019). Moreover, McFarlin et al. (2005) reported that exercise training improves NK-cell activity. PA might reduce the cytokine storm following infection with SARS-CoV-2. Regular PA or exercise facilitates the release of anti-inflammatory myokines (Marino et al., 2022); reduces chemokines, pro-inflammatory cytokines, and the pathogen load (Lowder et al., 2006; Sim et al., 2009; Warren et al., 2015); and promotes the balance of pro- and anti-inflammatory cytokines *via* renin-angiotensin system (RAS) modulation (Agarwal et al., 2011). Increased vagal tone resulting from high levels of PA also reduces pro-inflammatory cytokines (Tracey, 2009). In addition, aerobic exercise training decreases tissue inflammation by increasing antioxidant enzyme levels (superoxide dismutase and glutathione peroxidase) and mitochondrial biogenesis (Toledo et al., 2012).

COVID-19 patients had changes in the alveolar-capillary membrane (ACM) (Yan et al., 2020), resulting in an impairment of the diffusion capacity of the lungs (Yan et al., 2020). Aerobic exercise training improves the diffusing capacity for carbon monoxide during resting and exercise by modifying the ACM. Enhancing the density and affinity of adrenoreceptors in respiratory muscles through aerobic exercise may improve respiratory muscle function, bronchodilation, and airway secretion production, thereby improving lung function (Tret’yakov et al., 2020). The more preserved lung function and the sparing use of respiratory regulatory elements during stress increase the ability to mobilize efficiently when needed.

A strategy for COVID-19 treatment may be attenuating the risk of comorbidities associated with severity and mortality, such as obesity, diabetes mellitus, stroke, and coronary artery disease. Regular exercise increases maximal oxygen uptake, shifting high-risk patients into low-risk patients (Ahmed, 2020). The metabolic dysfunction, impaired immune response, and increased adipose-tissue inflammatory cytokine secretion associated with obesity can increase the risk of severe pneumonia in COVID-19 patients (Cai et al., 2020; Popkin et al., 2020). Aerobic exercise improves metabolic function and the immune response and decreases obesity, especially central obesity. Disruptions of the ACM in the lung tissues of obese and diabetic patients were observed (Foster et al., 2010). The increased thickness and lowered elasticity of the ACM impaired the diffusion capacity of the lungs (Foster et al., 2010). Reducing the risk factors associated with COVID-19 infection and severity may reduce mortality.

This study has several strengths. First, the systematic review and meta-analysis followed a standard protocol (Page et al., 2021). Second, the included studies were of high quality, denoted by their high NOS scores for quality assessment. Third, the included factors’ adjusted ORs are likely to be involved in the severity and mortality of infection in COVID-19 patients, for example, demographic variables, comorbidities, medical history, smoking status, and alcohol consumption.

There are some limitations to this study. First, it included only retrospective, cross-sectional, case-control, and nonrandomized controlled trials. Because COVID-19 is a newly emerging disease, studying the effects of regular PA requires long-term monitoring. Moreover, using a randomized controlled trial may be ethically inappropriate. Second, severity definitions were different among studies. Some included studies did not mention, some defined patients admitted to ICU and some represented hospitalization. Third, there are different patterns of PA, and exercise training was observed. Some of the included studies failed to report full details of the PA or exercise, for example, type, intensity, frequency, and time to perform an activity. This may be the source of the high heterogeneity in severity (I^2^ = 86%) and mortality (I^2^ = 85.0%) found in our study.

On the other hand, the forest plots showed that the studies agreed that PA might induce less severe illness and lower mortality in COVID-19 patients than PiA. This finding implies that engaging in regular PA, even in different patterns, has beneficial effects on the severity and mortality of COVID-19 patients. More studies on the impact of regular PA or exercise training are needed to draw definitive conclusions. Finally, the levels of PA and exercise were evaluated by self-reporting questionnaires, which may not directly reflect the actual performance of the patients. Therefore, for clinical application, the results should be carefully interpreted. Nevertheless, this study provided knowledge and understanding to medical professionals about the effects of regular PA on severity and mortality in COVID-19 patients.

A previous meta-analysis from a few studies showed that low and moderate-vigorous PA lowered mortality in COVID-19 patients (Rahmati et al., 2022). However, only one study (Lee et al., 2021) was included to show the lower ICU admission. The researchers suggested that levels of PA should be recommended. A recent study of pooled ORs adjusted by confounding factors concluded that PA ≥ 150 min/wk of moderate activity or ≥75 min/wk of vigorous activity showed a reduction in severity and mortality risk in COVID-19 patients. A strategy for COVID-19 treatment has been reported to prevent infection by SARS-CoV-2. The strategy involves preventing viruses from entering the body; blocking the binding between the virus and host cells; preventing new viral replication; and improving the immune response, anti-inflammation, antioxidants, and body functions. Possible explanations for why PA and exercise training reduce severity and mortality in COVID-19 patients include improvements in the systemic immune response, inflammatory cytokines, antioxidants, and lung function. Moreover, PA may reduce viruses entering the cells and lower some risk factors (eg, obesity, diabetes mellitus, and arterial hypertension) associated with severe outcomes for COVID-19 patients. However, lockdowns and restrictions due to the COVID-19 pandemic might increase barriers to engaging in regular PA. A tele-exercise program may be a strategy for promoting PA to prevent infection and reduce the severity and mortality of illness in COVID-19 patients.

## Conclusion

Regular PA can reduce the severity and mortality risk of COVID-19 patients, especially with ≥150 min/wk of moderate activity or ≥75 min/wk of vigorous activity.

## Data Availability

The raw data supporting the conclusions of this article will be made available by the authors, without undue reservation.

## Supplementary material

Supplementary Figure S1 Assessing publication bias by funnel plot: **(A)** effects of physical activity on mortality; **(B)** effects of physical activity on severity.

Supplementary Figure S2 Assessing publication bias by Egger’s test: **(A)** effects of physical activity on mortality; **(B)** effects of physical activity on severity. Abbreviations: CI, confidence interval; SND, standard.

Supplementary Table S1 Data search algorithm.

Supplementary Table S2 Excluded studies after assessment for eligibility.
