# Supplementary material for: Effects of physical activity on the severity of illness and mortality in COVID-19 patients: A systematic review and meta-analysis
Source: Front Physiol. 2022 Nov 8;13:1030568. doi: 10.3389/fphys.2022.1030568 (PMC9686861; doi:10.3389/fphys.2022.1030568)
Supplement: Supplementary file 2 [file Table1.docx]

**Supplementary Figure illustrations**

**Figure S1** Assessing publication bias by funnel plot: (a) effects of physical activity on mortality; (b) effects of physical activity on severity.

**Figure S2** Assessing publication bias by Egger’s test: (a) effects of physical activity on mortality; (b) effects of physical activity on severity. Abbreviations: CI, Confidence interval; SND, standard.

**Supplementary Table illustrations**

**Table S1** Data search algorithm.

**Table S2** Excluded studies after assessment for eligibility

**Table S1** Data search algorithm

| **Data base** | **Search strategy** | **Found** |
| --- | --- | --- |
| **Pubmed** | COVID* | 258,779 |
|  | (exercise) OR ("physical activity") | 596,969 |
|  | ("no exercise") OR ("no physical activity") | 1,880 |
|  | (severity) OR (mortality) | 2,996,797 |
|  | **(((COVID*) AND ((exercise) OR (physical activity))) AND ((no exercise) OR (no physical activity))) AND (((severity) OR (mortality))** | **1,420** |
| **Cochrane** | #1: (COVID*) | 11,793 |
|  | #2: (exercise) OR (physical activity) | 140,894 |
|  | #3: (no exercise) OR (no physical activity) | 57,752 |
|  | #4: (severity) OR (mortality) | 211,130 |
|  | **#1 and #2 and #3 and #4** | **363** |
| **Scopus** | (covid*) | 495,151 |
|  | (exercise OR "physical activity") | 2,126,373 |
|  | (no AND physical AND activity OR no AND exercise) | 279,496 |
|  | (severity OR mortality) OR (mortality) | 5,707,890 |
|  | **(COVID*) AND (exercise OR "Physical activity") AND (no physical activity OR no exercise) AND (severity OR mortality)** | **2,505** |
| **Science**  **Direct** | (COVID) | 136,159 |
|  | (exercise) OR (physical activity) | 2,738,323 |
|  | (no exercise) OR (no physical activity) | 2,474,789 |
|  | (severity) OR (mortality) | 2,504,439 |
|  | **((COVID) AND ((exercise) OR ("physical activity"))) AND (((COVID) AND ((no exercise) OR (no physical activity))) AND ((COVID) AND ((severity) OR (mortality)))** | **6,810** |
| **Embase** | #1: covid* | 265,879 |
|  | #2: "physical activity" OR exercise | 810,311 |
|  | #3: (no AND "physical activity") OR (no AND exercise) | 217,118 |
|  | #4: (severity) OR (mortality) | 2,843,191 |
|  | **#1 AND #2 AND #3 AND #4** | **320** |
| **OPENGREY.EU** | (COVID) | 1,793 |
|  | (exercise) OR (physical activity) | 4,826 |
|  | (“no exercise”) OR (“no physical activity”) | 5 |
|  | (severity) OR (mortality) | 2,023 |
|  | **(COVID) AND ((exercise) OR (physical activity)) AND ((“no exercise”) OR (“no physical activity”)) AND ((severity) OR (mortality))** | **1** |
| **ClinicalTrials.gov** | (COVID) | 8,431 |
|  | (exercise) OR (physical activity) | 19,388 |
|  | (no exercise) OR (no physical activity) | 50 |
|  | (severity) OR (mortality) | 5,663 |
|  | **(COVID) AND ((exercise) OR (physical activity)) AND ((“no exercise”) OR (“no physical activity”)) AND ((severity) OR (mortality))** | **0** |

**Table S2** Excluded studies after assessment for eligibility

| **Authors** | **Year** | **Title** | **Reasons for excluding** |
| --- | --- | --- | --- |
| Bliss et al. | 2022 | Impact of Exercise on Susceptibility and Severity of COVID-19 in Patients with Cancer: A Retrospective Study. | No full-text |
| Bielik et al. | 2021 | A Possible Preventive Role of Physically Active Lifestyle during the SARS-CoV-2 Pandemic; Might Regular Cold-Water Swimming and Exercise Reduce the Symptom Severity of COVID-19? | Inappropriate exposure grouping |
| de Souza et al. | 2021 | Association of physical activity levels and the prevalence of COVID-19-associated hospitalization. | Ineligible outcome |
| Gündoğdu  et al. | 2022 | Evaluation of the relationship between the disease severity and the level of physical activity in patients followed up with COVID-19 diagnosis. | Ineligible outcome |
| Gualano et al. | 2022 | Association between physical activity and immunogenicity of an inactivated virus vaccine against SARS-CoV-2 in patients with autoimmune rheumatic diseases. | Ineligible outcome |
| Kontopoulou  et al. | 2022 | Exercise Preferences and Benefits in Patients Hospitalized with COVID-19. | Ineligible outcome |
| Trapé et al. | 2021 | Effects of moderate-intensity intermittent hypoxic training on health outcomes of patients recovered from COVID-19: the AEROBICOVID study protocol for a randomized controlled trial. | Ineligible outcome |
| Tsushita et al. | 2022 | Survey of the effect of the SARS-CoV-2 outbreak on the job training members with high exercise intensity: Analysis of the factors causing infection spread and the effectiveness of the prevention measures. | Ineligible outcome |
| Güler et al. | 2021 | A survey on relationship between Gendarmerie Coast Guard Academy (GCGA) students' physical activity and COVID-19 infection. | Ineligible outcome |
| Chen et al. | 2022 | Causal relationship between physical activity, leisure sedentary behaviors  and COVID‑19 risk: a Mendelian randomization study | Ineligible study design |
| Li and Hua | 2021 | Modifiable lifestyle factors and severe COVID‑19 risk: a Mendelian randomisation study | Ineligible study design |
| Mohamed and Alawna | 2021 | The effect of aerobic exercise on immune biomarkers and symptoms severity and progression in patients with COVID-19: A randomized control trial. | Ineligible study design |
| Wang et al. | 2021 | Worldwide association of lifestyle-related factors and COVID-19 mortality. | Ineligible study design |
| Rahmati et al. | 2022 | Baseline physical activity is associated with reduced mortality and disease outcomes in COVID-19: A systematic review and meta-analysis. | Ineligible publication type |
| Clemente-Suárez et al. | 2022 | Physical activity and COVID-19. The basis for an efficient intervention in times of COVID-19 pandemic. | Ineligible publication type |
| Chesnut et al. | 2021 | Could diet and exercise reduce risk of COVID-19 syndemic? | Ineligible publication type |
| Khoramipour  et al. | 2021 | Physical activity and nutrition guidelines to help with the fight against COVID-19. | Ineligible publication type |
| Filgueira et al. | 2021 | The Relevance of a Physical Active Lifestyle and Physical Fitness on Immune Defense: Mitigating Disease Burden, With Focus on COVID-19 Consequences. | Ineligible publication type |
| Kushkestani  et al. | 2022 | SARS-COV-2 in Type 2 Diabetic Patients: Possible Roles of Exercise Training as a Medicine. | Ineligible publication type |
| Marino et al. | 2022 | Metabolic and inflammatory health in SARS-CoV-2 and the potential role for habitual exercise in reducing disease severity. | Ineligible publication type |
| Molanouri et al. | 2022 | Combined Effects of Exercise Training and Nutritional Supplementation in Cancer Patients in the Context of the COVID-19: A Perspective Study. | Ineligible publication type |
| Waluyo et al. | 2021 | Optimizing Early Rehabilitation Intervention: Insights from Different Outcomes in 2 Patients with Severe COVID-19. | Ineligible publication type |
| Filgueira et al. | 2021 | The Relevance of a Physical Active Lifestyle and Physical Fitness on Immune Defense: Mitigating Disease Burden, With Focus on COVID-19 Consequences. | Ineligible publication type |
| Després et al. | 2021 | Severe COVID-19 outcomes - the role of physical activity. | Ineligible publication type |
| Chastin et al. | 2021 | Effects of Regular Physical Activity on the Immune System, Vaccination and Risk of Community-Acquired Infectious Disease in the General Population: Systematic Review and Meta-Analysis. | Ineligible publication type |

(a)


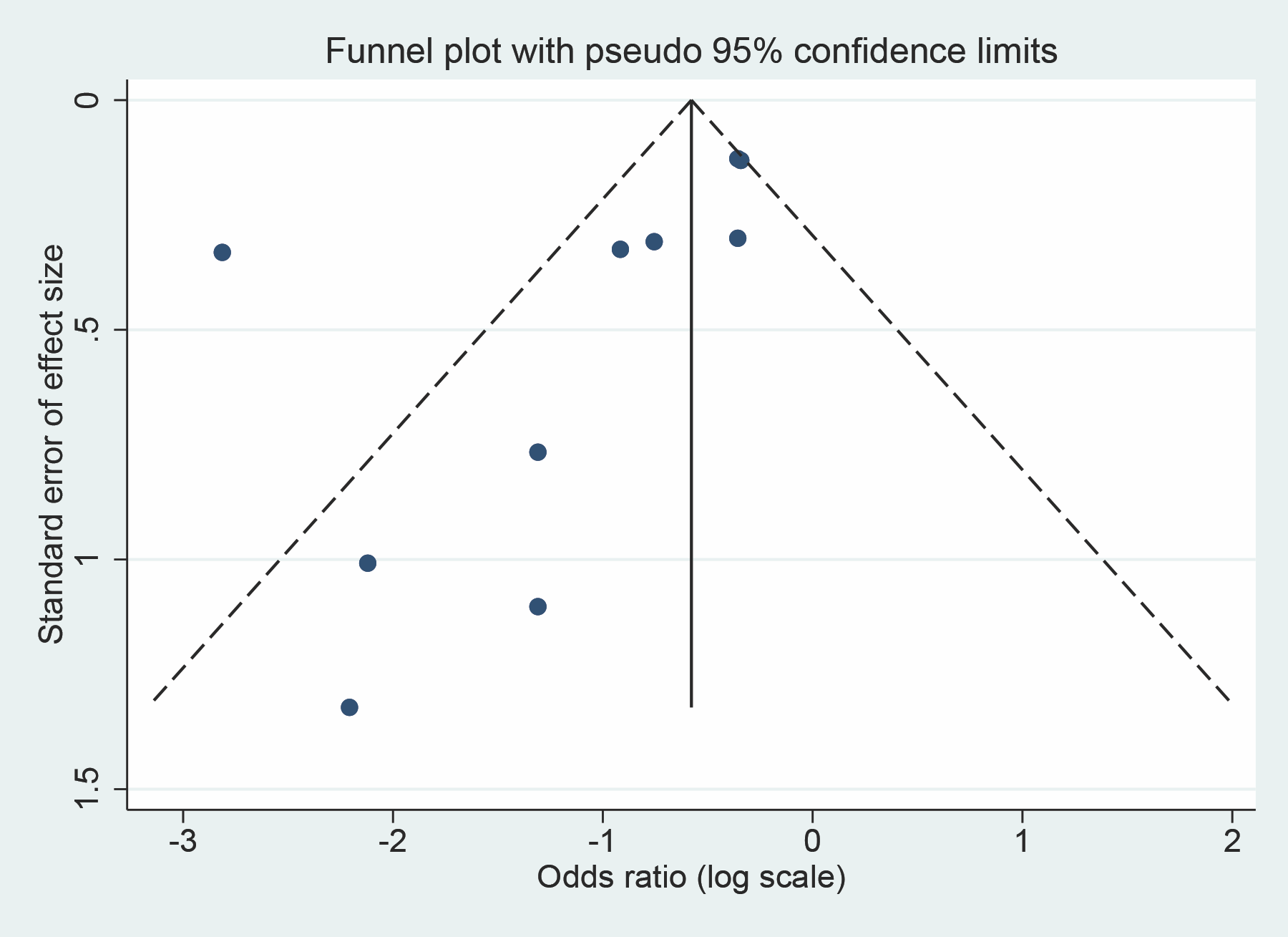


(b)


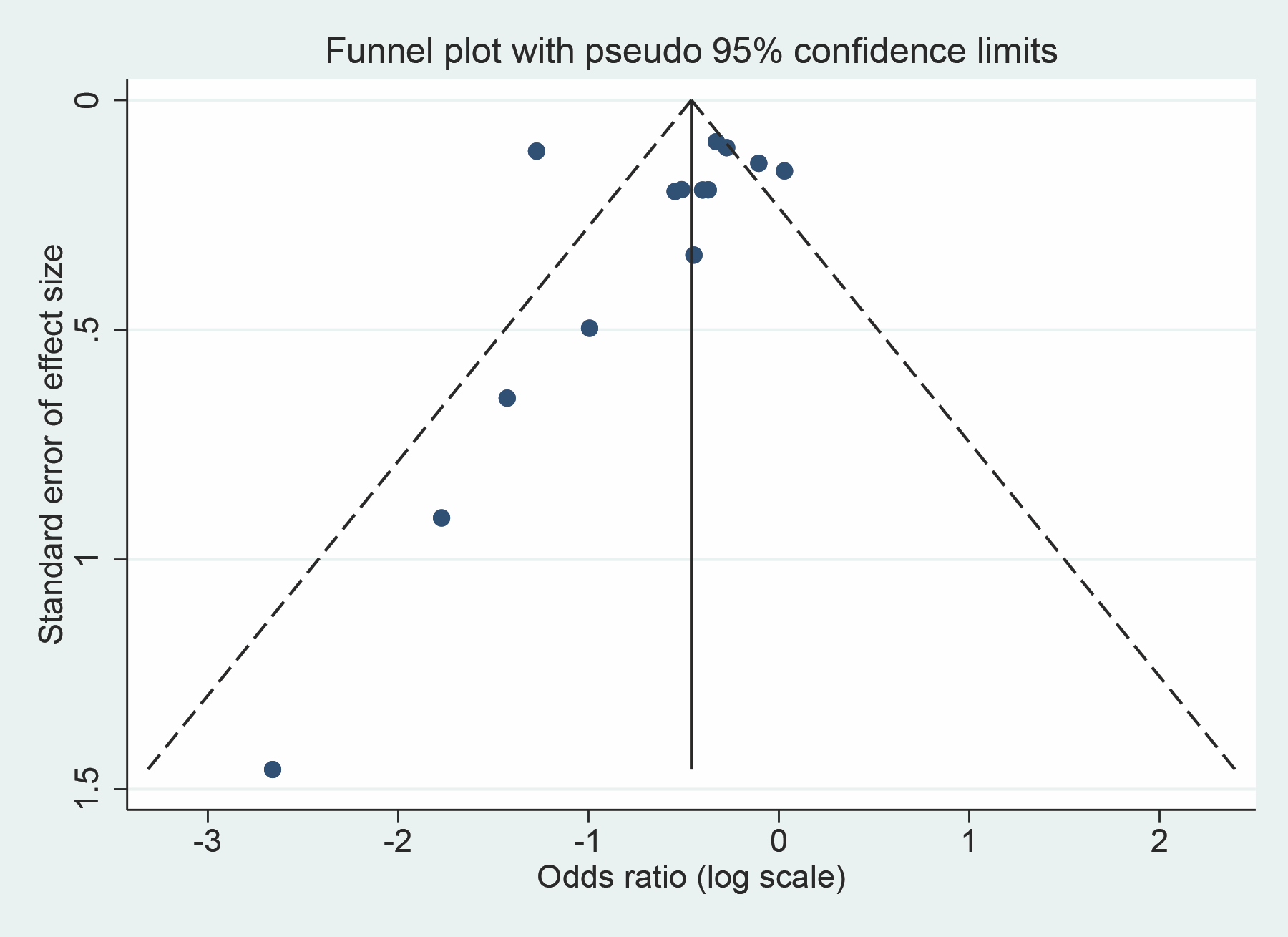


**Figure S1** Assessing publication bias by funnel plot: (a) effects of physical activity on mortality; (b) effects of physical activity on severity.

(a)


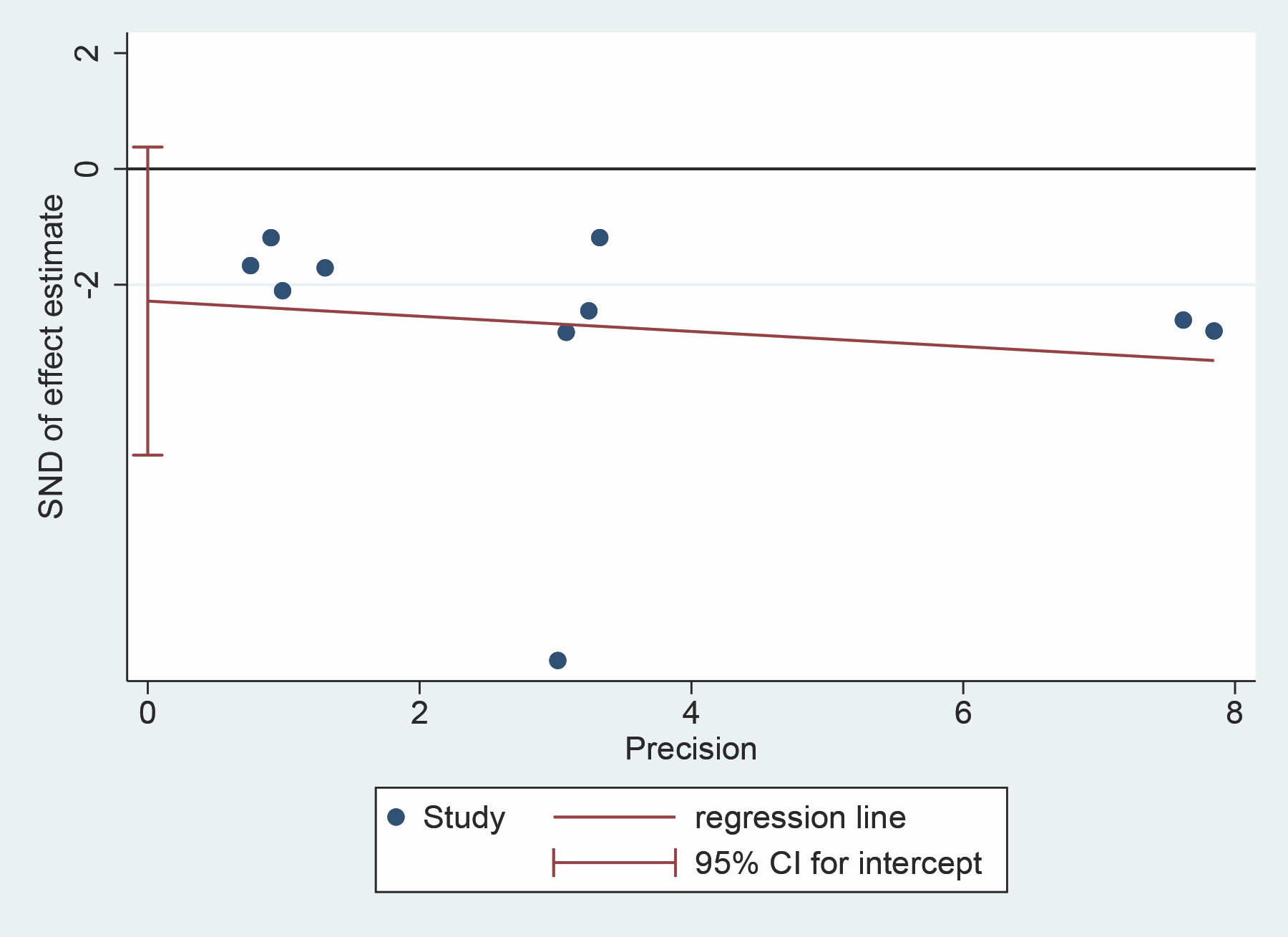


(b)


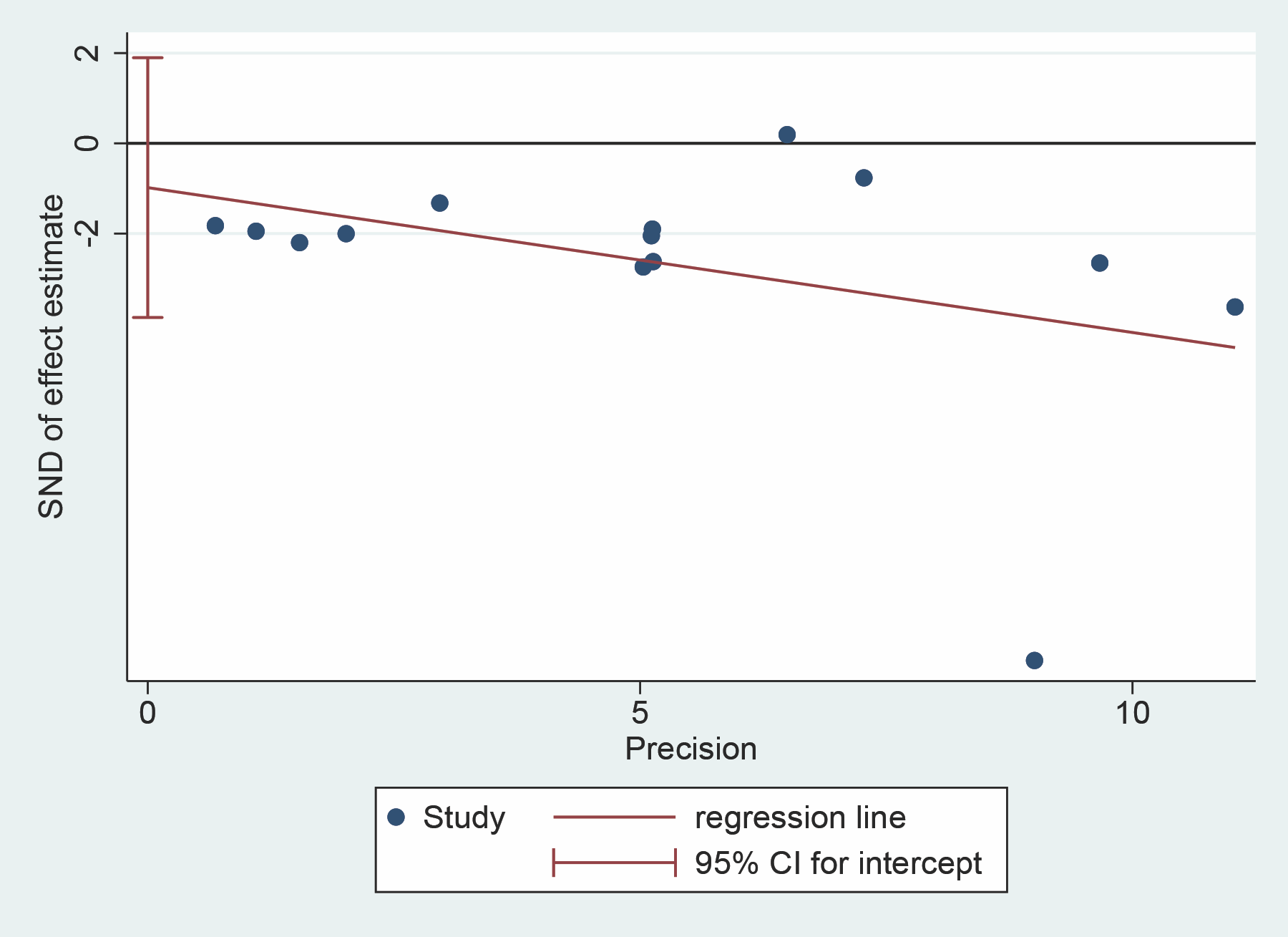


**Figure S2** Assessing publication bias by Egger’s test: (a) effects of physical activity on mortality; (b) effects of physical activity on severity. Abbreviations: CI, Confidence interval; SND, standard.
